# Temporal evolution of Sow herd age structure and its impact on the performance of Spanish commercial farms

**DOI:** 10.1186/s40813-025-00451-8

**Published:** 2025-06-19

**Authors:** S. Sanz-Fernández, C. Díaz-Gaona, J. Simões, J. C. Casas-Rosal, N. Alòs, L. Tusell, R. Quintanilla, Vicente Rodríguez-Estévez

**Affiliations:** 1 Departamento de Producción Animal, IC Zoonosis y Enfermedades Emergentes ENZOEM, Facultad de Veterinaria, Campus de Excelencia Internacional Agroalimentario (ceiA3), Universidad de Córdoba, Campus de Rabanales, Córdoba, 14071 España; 2 Department of Veterinary Science, CECAV-Animal and Veterinary Research Center, AL4AnimalS-Associate Laboratory for Animal and Veterinary Sciences, University of Trás-os-Montes e Alto Douro, Vila Real, 5000-801 Portugal; 3 Departamento de Matemáticas, Universidad de Córdoba, Avd. San Alberto Magno s/n, Córdoba, 14071 España; 4 Animal Breeding and Genetics Program, IRTA, Torre Marimon, Caldes de Montbui, 08140 Spain

**Keywords:** Census stability, Culling rate, Farm productivity, Herd management, Parity distribution

## Abstract

**Background:**

Efficient herd management is crucial for maintaining constant pig production. This study analysed the evolution of census structure over time and how productive performance variables related to farm efficiency are affected and evolved, using data from 427 Spanish commercial pig farms over three years (2020–2022). Farms were classified into three types of herd age structures based on the first coefficient of a quadratic function representing sow parity distribution. A longitudinal analysis was performed to evaluate changes in herd age structure and productivity over time, applying repeated measures ANOVA.

**Results:**

The herd age structure types in 2020 were: HS1 (with a downward-concave trend), HS2 (with a trend close to a straight line), and HS3 (with an upward-concave trend). HS1 farms had the highest productivity over time, maintaining superior performance compared to HS2 and HS3 (*p* < 0.01), with 31.4 piglets weaned per sow per year in 2021 and 30.9 in 2022. However, HS1 farms showed moderate consistency in herd parity structure over time, with 48.6% remaining in the same group in 2021 and 43% in 2022. HS2 farms showed the greatest herd parity structure stability over time, with 60.1% and 54.5% of farms remaining in this group in 2021 and 2022, respectively. HS3 farms were the least stable, with only 29% retaining their classification by 2022, and had the lowest productivity.

**Conclusions:**

Classifying farms by herd age structure provides valuable insights into how parity distribution influences farm productivity over time and how herd parity structure evolves. HS1 farms achieved the best productivity over the study but require specific management practices to maintain their stability. HS2 remained with the most stable herd structure over time, with intermediate productivity, while HS3 farms showed the worst stability and performance. Thus, HS1 is recommended as the optimal herd age structure for maximizing productivity in the short and medium term. Further research should focus on identifying specific management factors to optimize productivity and ensure long-term herd structure stability of HS1.

## Background

The proportion of sows according to cycles or parities is crucial, as important parameters such as prolificacy, fertility, or piglet survival experience significant variations throughout reproductive cycles [1–5]. Therefore, efficient management of herd age structure is a fundamental pillar for maintaining constant pig production and improving the productive efficiency of farms over time [6].

Other factors such as the replacement or culling rate emerge as determining factors that shape the dynamics of herd age structure [7, 8]. To this respect Jalvingh et al. [9] highlighted the importance of studying sow herd dynamics over time to maintain a constant farm production.

Thus, stability in herd age structure dynamics becomes essential to ensure long-term farm productivity [10]. Several studies have examined models for managing sow herd dynamics [8, 10–14], identifying the need for research that supports decision-making adapted to the real-life dynamics of farm herds.

A previous study [15] classified farms according to their herd age structure in three groups, concluding that farms with a higher proportion of sows in intermediate parities (3rd to 5th) show better productive results over a year. A question that arose from this study was whether this structure, while productive in the short term, is the most suitable in the medium or long term and whether it can remain stable over time.

Considering the current average of 2.4 litters per sow per year [16, 17], a primiparous sow reaches its fifth parity within two years. This dynamic implies that the herd age structure of a given year can undergo significant changes in the following two years. For example, a herd age structure characterized by a higher percentage of sows from 3rd to 5th parities, without an adequate replacement and culling policy would turn to a structure with a predominance of sows of ≥ 6th parities within just one year. Thus, while initially productive [18], that structure can become least productive after one year [7].

In this context, there is a need to study how herd age structures evolve over time. The aim of this study is to determine which type of herd age structure is most suitable in the short and medium term (up to three years) and to evaluate how productivity and herd age structure simultaneously evolve in pig farms. Furthermore, this study seeks to provide a deeper understanding of the influence of herd age structure on reproductive efficiency, highlighting the importance of maintaining a stable herd to avoid annual fluctuations.

## Materials and methods

The dataset analysed in this study originates from the BDporc^®^ databank, thanks to a collaborative agreement between the Institute of Agrifood Research and Technology (IRTA) and the Department of Animal Production of the University of Córdoba. Farms having complete and continuous records of census as well as litters for at least six parities over the analysed period were selected for the study. Consequently, the study included data from 427 Spanish commercial breeding farms (no Iberian breed sows were included) (Table 1), for which population structure (census) data and productive parameters were recorded over three consecutive years (2020, 2021, and 2022). All the analysed farms operated under a batch farrowing system, either on a weekly or three-weekly basis. No information was available regarding the health status of the farms.

**Table 1:** Total number of farms, sows, and litters included in the study

Year	Number of farms within BDporc	Number of farms included (%)	Number of sows included	Number of litters included
2020	631	427 (67.7%)	610,092	1,287,941
2021	588	427 (72.6%)	622,719	1,289,640
2022	531	427 (80.4%)	616,676	1,262,531

The productive parameters collected for each farm were: mean number of sows on the farm; replacement rate; piglets weaned per sow per year (PWSY); age of sows at culling (months); farrowings per culled sow; total number of piglets weaned per culled sow throughout her life; farrowings per sow and year; farrowing rate; percentage of sow returning to oestrus; weaning-to-first-service interval (WSI); weaning-to-oestrus interval (WOI); weaning-to-conception interval (WCI); number of total born (TB), born alive (BA), stillborn (SB) and weaned (W) piglets; and mortality rate of BA piglets at weaning (i.e., during lactation). Although data collection was carried out on a quarterly basis, the analyses in this study were performed using the annual average values from each farm.

Regarding the distribution of sows by parity, BDporc groups sows with 8 or more parities into a single census group to homogenize the studied sample.

### Modelling and classifying the farm’s herd age structure over three years

Following the methodology proposed by Sanz-Fernández et al. [15], farms were classified into three groups based on the herd age structure. This classification relies on a quadratic function that models herd age structure as a parabola, defined by the equation: f(x) = ax^2^ + bx + c. The dependent variable is the percentage of sows at each parity, and the independent variable is the number of parity (from 1 to ≥ 8 parities).

A separate quadratic regression was fitted for each of the 427 farms, allowing an independent classification based on herd age structure. Specifically, the classification method is based on the value of the “a” coefficient of the quadratic function obtained by graphically representing each farm. The “a” coefficient defines the graph’s orientation, determining the function’s curvature and whether the parabola opens upwards or downwards [19]. Additionally, the absolute value of “a” indicates the magnitude of the parabola’s curvature and its direction, effectively characterizing the herd’s census structure [15]. To calculate the “a” coefficient, Python Editor V.3.10.10, Pyzo V.4.12.8 *(Python Software Foundation*,* Wilmington*,* DE*,* USA)*, was used, employing the least squares method for the quadratic functions of the census distribution of the 427 farms in 2020, 2021, and 2022.

**Fig. 1:**
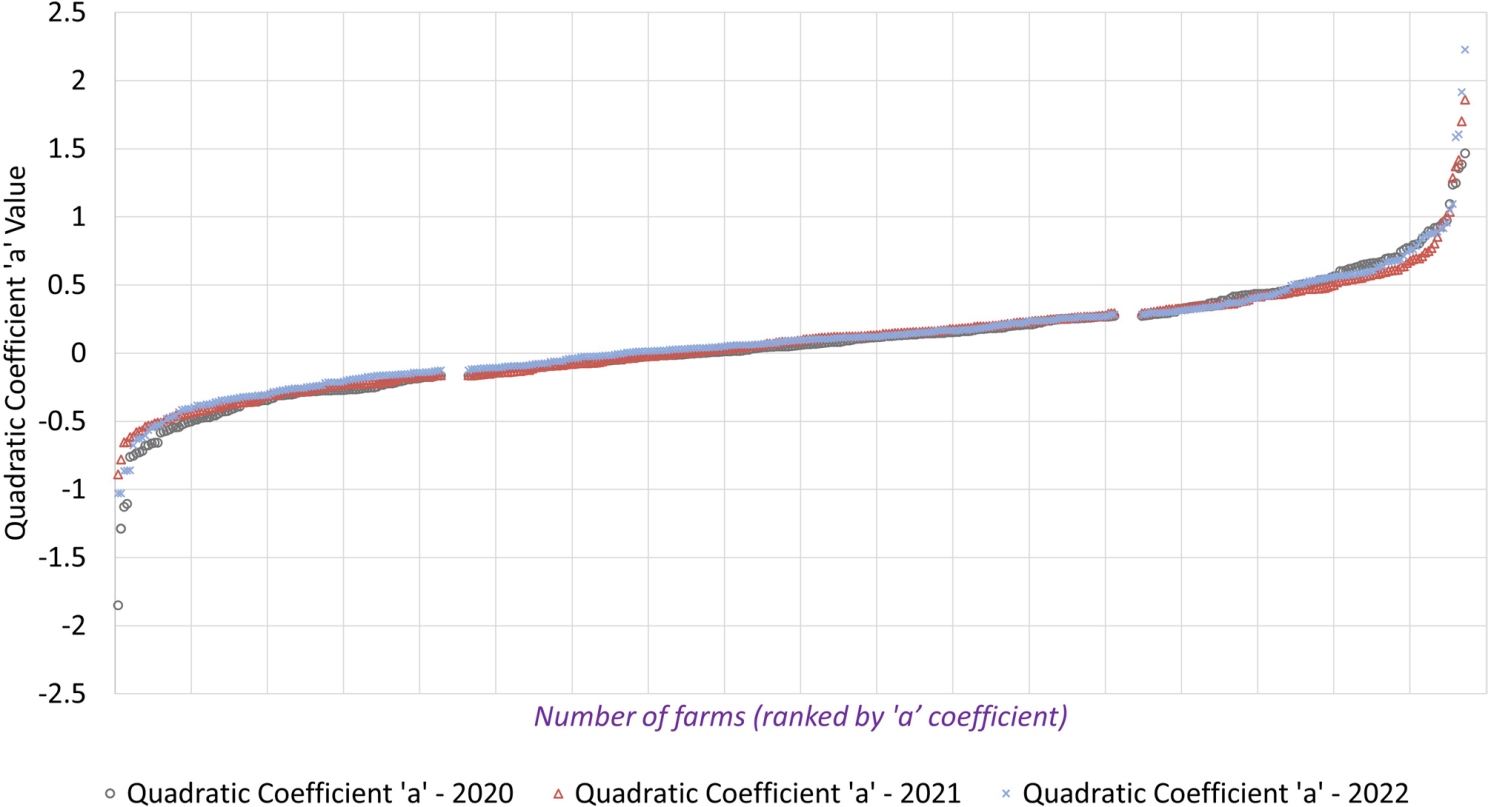
Annual distribution of the “a” coefficient used for herd age structure classification (*N* = 427 farms per year). Abbreviations: HS1: Herd structure type 1 (includes the 25% of farms with the lowest and most negative values for the “a” coefficient within each year); HS2: Herd structure type 2 (includes the 50% of farms with intermediate values (close to zero) of the “a” coefficient); HS3: Herd structure type 3 (includes the 25% of farms with the highest and most positive values of the “a” coefficient values within each year)

According to that coefficient three types of herd age structure were established annually (Fig. 1): type 1 (HS1) includes the 25% of farms with the lowest and most negative values for the “a” coefficient within each year. This structure is characterized by maintaining a high proportion of sows in the intermediate parities (3rd to 5th ). Type 2 (HS2) includes the 50% of farms with intermediate values (close to zero) of the “a” coefficient. This structure is characterized by a constant decline in the proportion of sows across parities. Type 3 (HS3) includes the 25% of farms with the highest and most positive values of the “a” coefficient values within each year. This structure is characterized by a higher proportion of sows in the later and earliest parities compared to the other types of herd age structures.

Additionally, the coefficient of determination (R²) and the root mean square error (RMSE) of the quadratic function were computed for each herd age structure group.

### Statistical analysis

Statistical analyses were conducted using IBM SPSS 22 software *(IBM Corp.*,* Armonk*,* NY*,* USA)*. First, according to the type of herd age structure over the three years, descriptive statistics of BA and W piglets for each parity and the sow percentage by parity were calculated. Second, descriptive statistics of the aforementioned production parameters of all farms were calculated for the three years of study. Normality of analysed production variables was assessed; all variables met the normality criteria stablished by Kline [20], excepting WSI, WOI, and WCI, which exhibited kurtosis values above 8. Therefore, the results obtained for these variables in the following analyses should be interpreted with caution.

#### Repeated measures ANOVA for productive performance analysis

A repeated measures analysis of variance (ANOVA) was conducted to evaluate the variation of the production variables in farms across three types of herd structure over three consecutive years. Therefore, the ANOVA considered the following factors: the analysed year (three levels: 2020, 2021 and 2022), the herd structure classification in year 2020 (three levels: HS1, HS2 and HS3), and the interaction between these two factors. Additionally, a pairwise mean comparison of productive performance variables across years and across herd structures was performed by Tukey’s HSD test. The alpha level for determining significance of these factors was set at 0.05.

In cases where significant differences were observed, the Partial Eta Squared (η²) was calculated as a measure of effect size. This statistic quantifies the proportion of variance in the dependent variable (production parameters) explained by the independent variable, helping to assess the magnitude of the observed differences [21]. According to Cohen [22], effect size levels are classified as small (η^2^ = from 0.01 to < 0.06), medium (η^2^ = from 0.06 to < 0.14) and large (η^2^ ≥ 0.14).

#### Temporal evolution of herd age structure

Finally, to assess which type of herd age structure remains most stable over time, a contingency table analysis was performed along with the determination of the Kappa coefficient (κ). The Kappa statistic measures the agreement between classifications over time, beyond what would be expected by chance. Specifically, it evaluates the consistency of farms’ classifications (HS1, HS2, or HS3) from year to year. A κ value close to 1 indicates strong agreement, meaning that most farms retained their classification over time, whereas a κ value near 0 suggests frequent changes, indicating weak agreement. This analysis aimed to identify which herd age structure type remained the most stable by quantifying the proportion of farms that changed categories over the three years of study.

## Results

### Types of herd age structure during years

**Fig. 2:**
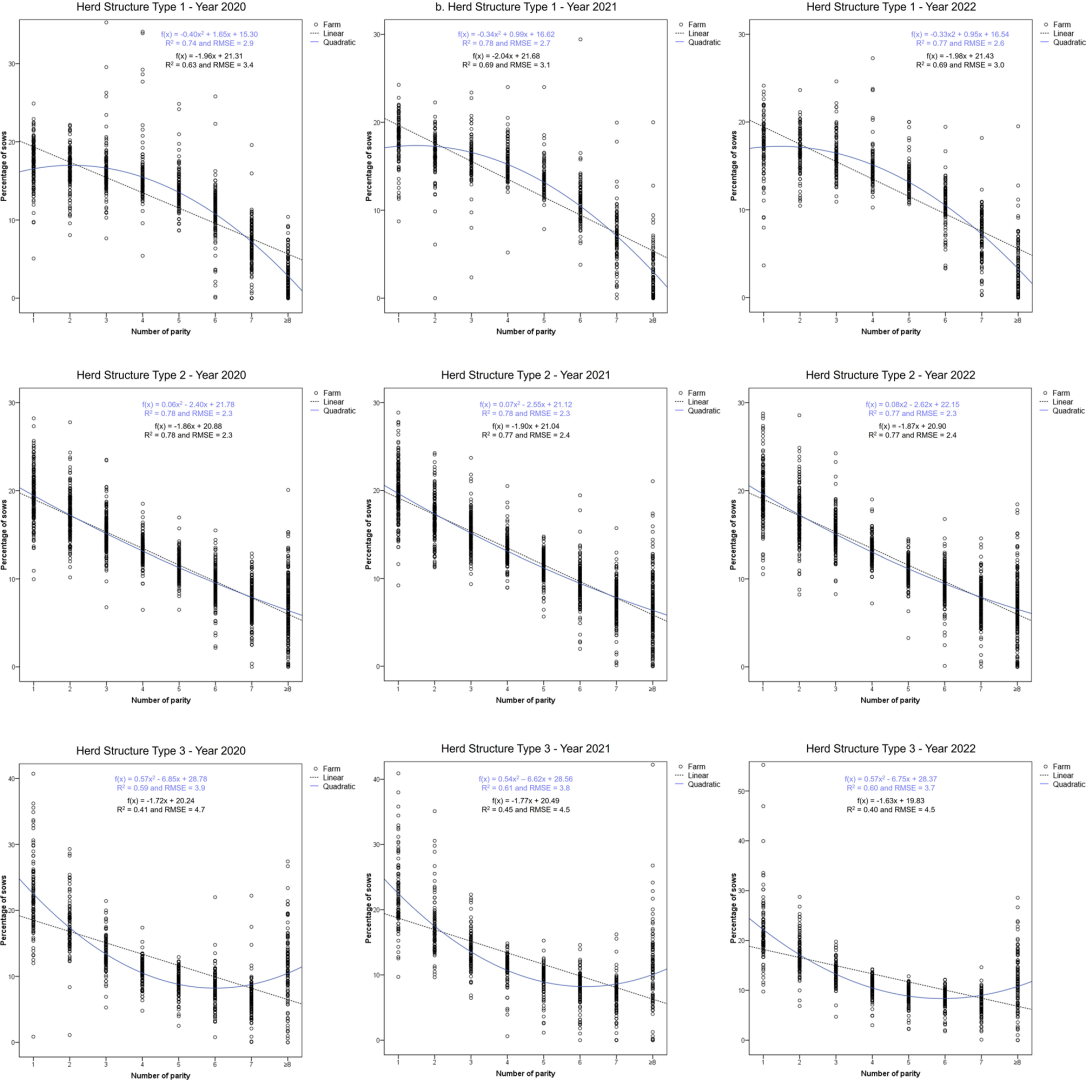
Linear and quadratic function fitting for herd structure types in 2020, 2021 and 2022 (*N* = 427 farms per year). The mean of each cluster of data points at each parity can be found in Table 2

The quadratic function fitted to the sow parity distribution in 2020, 2021, and 2022 are shown in Fig. 2. Table 2 shows the mean percentage of sows at each parity and the mean BA and W piglets per parity for these three groups of herd age structure over the 3 years of study.

**Table 2:** Descriptive statistics for the Sow distribution and prolificacy for piglets born alive and weaned at each parity according to the groups of herd age structures in the years 2020, 2021 and 2022 (*N* = 427)

			Parity 1	Parity 2	Parity 3	Parity 4	Parity 5	Parity 6	Parity 7	Parity ≥ 8
Year 2020	Herd Structure Type 1 (a25%) (*N* = 107)	Mean percentage of sows	17.2	16.4	16.1	15.4	14.0	11.3	7.1	2.6
		Number of piglets born alive	14.4	14.8	15.4	15.3	15.0	14.6	13.5	12.1
		Number of piglets weaned	13.0	13.1	13.0	12.9	12.7	12.6	12.0	10.9
	Herd Structure Type 2 (a50%) (*N* = 213)	Mean percentage of sows	19.4	17.2	15.2	13.3	11.4	9.4	7.3	6.8
		Number of piglets born alive	13.9	14.4	15.0	15.1	14.8	14.4	13.8	13.3
		Number of piglets weaned	12.6	12.6	12.4	12.3	12.1	11.9	11.7	11.7
	Herd Structure Type 3 (a75%) (*N* = 107)	Mean percentage of sows	22.1	17.7	13.6	10.8	9.2	8.1	6.8	11.7
		Number of piglets born alive	13.7	14.2	14.8	14.8	14.5	14.1	13.6	12.6
		Number of piglets weaned	12.1	12.3	12.2	12.1	12.0	11.9	11.7	11.3
Year 2021	Herd Structure Type 1 (a25%) (*N* = 107)	Mean percentage of sows	18.1	16.3	15.9	15.4	13.6	11.0	7.0	2.7
		Number of piglets born alive	14.1	14.6	15.4	15.4	15.1	14.6	13.9	12.5
		Number of piglets weaned	12.7	12.9	12.9	12.8	12.7	12.6	12.5	10.9
	Herd Structure Type 2 (a50%) (*N* = 213)	Mean percentage of sows	19.8	17.0	15.1	13.3	11.4	9.4	7.3	6.6
		Number of piglets born alive	14.2	14.7	15.3	15.3	15.0	14.6	14.1	13.4
		Number of piglets weaned	12.7	12.6	12.6	12.4	12.3	12.2	12.1	11.8
	Herd Structure Type 3 (a75%) (*N* = 107)	Mean percentage of sows	22.3	17.3	13.8	11.1	9.3	8.1	7.0	11.1
		Number of piglets born alive	14.0	14.5	15.1	15.1	14.8	14.4	13.7	12.9
		Number of piglets weaned	12.2	12.5	12.3	12.2	12.0	12.0	11.5	11.3
Year 2022	Herd Structure Type 1 (a25%) (*N* = 107)	Mean percentage of sows	17.5	16.7	16.3	15.1	13.5	10.6	7.1	3.2
		Number of piglets born alive	14.2	14.7	15.2	15.2	15.0	14.6	14.2	12.2
		Number of piglets weaned	12.7	12.9	12.8	12.7	12.6	12.7	12.6	10.7
	Herd Structure Type 2 (a50%) (*N* = 213)	Mean percentage of sows	19.6	17.0	15.3	13.2	11.1	9.4	7.4	6.9
		Number of piglets born alive	14.2	14.9	15.3	15.3	14.9	14.6	14.0	13.3
		Number of piglets weaned	12.3	12.5	12.4	12.2	12.1	12.1	11.9	11.6
	Herd Structure Type 3 (a75%) (*N* = 107)	Mean percentage of sows	21.8	17.3	13.5	10.8	9.1	8.3	7.4	11.8
		Number of piglets born alive	14.2	14.7	15.2	15.2	14.9	14.4	13.9	12.9
		Number of piglets weaned	12.3	12.3	12.2	12.0	12.0	11.9	11.6	11.0

HS1 farms maintain a high proportion of sows in the intermediate reproductive cycles. These farms had approximately 45% of their sows between the 3rd and 5th parities in the three analysed years, which are the parities with the highest prolificacy in terms of BA piglets (Table 2). Thus, the obtained quadratic function for these farms shows a concave-downward trend curve in the three analysed years (Fig. 2). The R² and RMSE values of the estimated functions indicated a better fit in 2022 and 2021 compared to 2020.

HS2 farms result in an average and relatively constant decrease of around 2% sows from one parity to the next over the three years of study, starting from a slightly higher proportion (around 19%) of first-parity sows than HS1 (Table 2). Thus, the quadratic function for these farms shows a trend curve that is close to a straight line, with minimal variations across the years (Fig. 2).

Lastly, HS3 farms were those having a highest proportion of sows in later cycles, which are the parities with the lowest prolificacy in terms of piglets BA (Table 2), as well as a higher proportion of primiparous sows. On average, these farms showed more than 18% sows in their 6th or higher parity and over 39% in their 1st and 2nd parities across the three analysed years. Thus, the obtained quadratic function for these farms shows an upward concave trend curve (Fig. 2), with quadratic functions showing a similar fit to the census structure across years, though with a lower R² compared to HS1 and HS2 farms.

### Evolution of productive parameters over the years

The descriptive statistics of the productive variables associated with farm performance in 2020, 2021, and 2022 are shown in Table 3. The average annual productivity of the dataset over the three years reached 30.03 PWSY, with the highest annual productivity in 2021 (30.24 PWSY).

**Table 3:** Descriptive statistics for the productive variables of the commercial farms studied from 2020 to 2022 (*N* = 427)

	Year 2020		Year 2021		Year 2022		*p*-value
	Mean	SE	Mean	SE	Mean	SE	
Mean number of sows on the farm	1428.79	56.82	1458.36	57.51	1444.21	57.09	0.935
Replacement rate (%)	47.33	0.61	47.99	0.56	46.91	0.73	0.478
Number of piglets weaned per sow per year	30.12^a^	0.16	30.24^a^	0.16	29.72^b^	0.15	0.049
Culled sow age (months)	32.64^a^	0.22	32.26^a^	0.22	31.52^b^	0.20	0.001
Farrowings per culled sow	4.55^a^	0.04	4.45^a^	0.04	4.27^b^	0.04	< 0.001
Total number of piglets weaned per culled sow	55.31^a^	0.58	54.44^a^	0.55	52.44^b^	0.52	0.001
Farrowings per sow and year	2.43^a^	0.00	2.43^a^	0.00	2.42^b^	0.00	< 0.001
Farrowing rate (%)	85.23^ab^	0.26	85.65^a^	0.26	84.75^b^	0.28	0.049
Percentage of sows return to oestrus	13.41	0.25	12.67	0.25	13.17	0.28	0.127
Weaning-to-first-service interval (days)	6.05^b^	0.08	6.25^ab^	0.10	6.40^a^	0.10	0.024
Weaning-to-oestrus interval (days)	4.85	0.03	4.85	0.03	4.86	0.03	0.965
Weaning to conception interval (days)	9.08^b^	0.15	9.26^b^	0.17	10.17^a^	0.21	< 0.001
Number of piglets total born per litter	15.76^b^	0.10	15.98^ab^	0.10	16.11^a^	0.09	0.031
Number of piglets born alive (BA) per litter	14.44	0.08	14.64	0.08	14.64	0.08	0.122
Number of piglets still born per litter	1.33^b^	0.02	1.35^b^	0.02	1.47^a^	0.03	< 0.001
Number of piglets weaned per litter	12.36	0.07	12.43	0.06	12.29	0.06	0.295
Mortality rate of BA piglets at weaning (%)	14.27^b^	0.22	14.93^b^	0.23	15.90^a^	0.25	< 0.001

Different superscript letters ^(a-b)^ indicate differences among years (*p* < 0.05, Tukey HSD test). Abbreviations: SE = Standard error

Although some improvements were observed between 2020 and 2021, including an increase in farrowing rate (+ 0.43%) and prolificacy (+ 0.22 TB and + 0.20 BA piglets), as well as a decrease in the percentage of sows returning to oestrus (-0.74%), no statistically differences were found between these two years.

In contrast, in 2022, the annual productivity showed worse average results, with a decrease of PWSY compared to 2020 (-0.4) and 2021 (-0.5) (*p* < 0.05). These poorer results in 2022 may be due to the average increase in lactation mortality (+ 1.63%; *p* < 0.001), along with an increase in the number of stillborn piglets per litter (+ 0.15; *p* < 0.001). Regarding sow longevity, culled sow age decreased in 2022 compared to previous years (*p* < 0.01), and a similar pattern was observed for farrowings per culled sow, which also declined over time (*p* < 0.001).

Finally, reproductive efficiency indicators showed a progressive worsening over the years. WSI increased in 2022 compared to 2020 (*p* < 0.05), as did the WCI (*p* < 0.001), suggesting a decline in reproductive efficiency over time.

### Effect of year and herd age structure on productive performance

Results from the repeated measures ANOVA (Table 4) indicated that most productive variables (excepting replacement rate and WOI) showed differences across the three years of study (*p* < 0.01). Regarding the magnitude of these differences over the years, medium effect sizes of year effect were observed for the number of stillborn piglets and the mortality rate during lactation (η^2^ = 0.13 in both cases). Additionally, prolificacy (TB piglets) and the yearly number of farrowings per sow also showed medium effect sizes (η^2^ = 0.10 in both cases). Conversely, other variables showing an evolution over the years such as PWSY, number of BA piglets or farrowing rate, presented small effect sizes in this variation (η^2^ = 0.05, 0.04, and 0.02, respectively).

**Table 4:** Results from the repeated measures ANOVA of productive variables: significance and effect size of year and herd structure factors, and their interaction. The mean comparison of productive variables within the years 2021 and 2022 across the three farm herd structures in 2020 is also presented (*N* = 427)

Productive variables	Within-Subjects effects		Between-Subjects effects		Analysis of Interaction		Mean per year and herd structure^3^						
	Year (2020, 2021, 2022)		Herd structure^2^ (HS1, HS2, HS3)		Year*Herd structure interaction		Year 2021			Year 2021			
	*P*-value	Effect size	*P*-value	Effect size	*P*-value	Effect size	HS1^2^	HS2^2^	HS3^2^		HS1^2^	HS2^2^	HS3^2^
Mean number of sows on the farm	< 0.001	0.02	0.077	0.01	0.111	0.01	1303.55	1426.47	1676.64		1288.65	1417.92	1652.09
Replacement rate (%)	0.308	0.01	0.120	0.01	0.017	0.01	48.2	47.99	47.8		45.78	47.2	47.47
Number of piglets weaned per sow per year	< 0.001	0.04	< 0.001	0.06	0.621	0.00	31.41^a^	30.06^b^	29.44^b^		30.86^a^	29.60^b^	28.81^b^
Culled sow age (months)	< 0.001	0.04	0.984	0.00	< 0.001	0.03	32.36	32.26	32.16		32.01	31.62	30.81
Farrowings per culled sow	< 0.001	0.07	0.165	0.01	< 0.001	0.03	4.57	4.44	4.35		4.46^a^	4.28^a^	4.04^b^
Number of piglets weaned per culled sows	< 0.001	0.05	< 0.001	0.04	< 0.001	0.03	58.11^a^	53.82^b^	52.01^b^		56.98^a^	52.21^b^	48.35^c^
Farrowings per sow and year	< 0.001	0.10	0.041	0.01	0.316	0.01	2.44	2.44	2.42		2.42	2.42	2.4
Farrowing rate (%)	< 0.001	0.02	< 0.001	0.04	< 0.001	0.02	86.91^a^	85.57^ab^	84.56^b^		85.70^a^	84.84^ab^	83.62^b^
Percentage of sows return to oestrus	< 0.001	0.01	0.001	0.03	< 0.001	0.02	11.81^b^	12.6^ab^	13.69^a^		12.5	12.99	14.19
Weaning-to-first-service interval (days)	< 0.001	0.04	< 0.001	0.03	0.006	0.02	5.87^b^	6.14^b^	6.85^a^		6.12^b^	6.23^b^	7.01^a^
Weaning-to-oestrus interval (days)	0.750	0.00	0.011	0.02	0.154	0.01	4.80^b^	4.78^b^	5.03^a^		4.78^b^	4.82^ab^	5.02^a^
Weaning to conception interval (days)	< 0.001	0.07	< 0.001	0.03	0.093	0.01	8.61^b^	9.27^ab^	9.89^a^		9.30^b^	10.04^ab^	11.29^a^
Number of piglets total born	< 0.001	0.10	0.021	0.02	0.522	0.00	16.35^b^	15.96^ab^	15.66^a^		16.52^b^	16.05^ab^	15.82^a^
Number of piglets born alive (BA) per litter	< 0.001	0.05	0.006	0.02	0.425	0.00	14.99^a^	14.60^ab^	14.37^b^		15.04^a^	14.59^ab^	14.34^b^
Number of piglets still born per litter	< 0.001	0.13	0.903	0.00	0.117	0.01	1.36	1.36	1.29		1.49	1.46	1.48
Number of piglets weaned per litter	< 0.001	0.02	< 0.001	0.05	0.681	0.00	12.89^a^	12.33^b^	12.17^b^		12.73^a^	12.22^b^	11.99^b^
Mortality rate of BA piglets at weaning (%)	< 0.001	0.13	0.077	0.01	0.191	0.01	13.89^b^	15.34^ab^	15.16^a^		15.28	16.0	16.33

^1^ Effect sizes are reported as partial eta squared (η²)

^2^ Abbreviations: HS1 = Herd structure type 1 in 2020; HS2 = Herd structure type 2 in 2020; HS3 = Herd structure type 3 in 2020

^3^ Different superscript letters ^(a, b, c)^ indicate differences between herd structure groups within the same year (*p* < 0.05, Tukey HSD test)

The analysis of between-subject variables, i.e., the effect of the herd age structure type on the production parameters when removing the temporal factor, reveals that there are differences between farms of different herd age structure type for almost all productive variables (*p* < 0.05). However, the effect size of the herd age structure type was moderate, with annual productivity (PWSY) showing the greatest magnitude of differences between herd age structure types in 2020 (η^2^ = 0.06).

Additionally, no interaction were found between the evolution over time and the type of herd age structure for the main variables related to annual productivity: PWSY, number of farrowings per sow and year, and prolificacy (both TB and BA piglets per parity) (*p* > 0.05), suggesting a similar evolution of these variables over the years in the three groups of farms (HS1, HS2, and HS3). However, interactions were observed for replacement rate, farrowing rate, percentage of sows returning to oestrus, and WSI (*p* < 0.05). The same applies to variables related to sow longevity (culled sow age and farrowings per culled sow) and their lifetime productivity (number of piglets weaned per culled sow), indicating that the time effect on these parameters differed depending upon the type of herd age structure. Nevertheless, all differences explained by these interactions had a small effect size (η^2^ ≤ 0.03).

Finally, the mean productive parameters in 2021 and 2022 differed among farms based on their herd age structure in 2020. In terms of annual productivity, HS1 farms, characterized by a slightly concave-downward trend curve and a higher proportion of sows in the intermediate parities, continued showing superior performance in 2021 and 2022 (31.41 and 30.86 PWSY) when compared to HS2 (30.06 and 29.60 PWSY) and HS3 farms (29.44 and 28.81 PWSY) (*p* < 0.01). Similarly, farms with HS1 in 2020 had the highest farrowing rate in the two following years, 2021 (86.91%) and 2022 (85.70%), compared to the other types of herd age structure (*p* < 0.05). Additionally, the WSI, WOI, and WCI intervals were consistently more favourable in HS1, indicating better reproductive efficiency (less non productive days and higher fertility), especially compared to HS3 (*p* < 0.05).

Regarding prolificacy (both TB and BA piglets), it also remained higher in farms classified as HS1 in 2020, especially compared to HS3 farms (*p* < 0.05); while HS2 farms had intermediate prolificacy. Likewise, the mortality of piglets at weaning was higher in HS3 farms in 2020 and 2021 mainly when compared to HS1 farms (*p* < 0.05).

The variables related to sow longevity (culled sow age and farrowings per culled sow) did not show differences between herd age structure types in 2020. However, HS3 farms, characterized by a higher proportion of sows with ≥ 6 parities, showed increased sows longevity; although, this pattern changed in 2021, when these variables were similar among the three herd age structures. Conversely, in 2022, farms with HS1 in 2020 had more farrowings per culled sow than HS3 farms (4.46 vs. 4.04; *p* < 0.01).

### Evolution of the herd age structure over three years

**Fig. 3:**
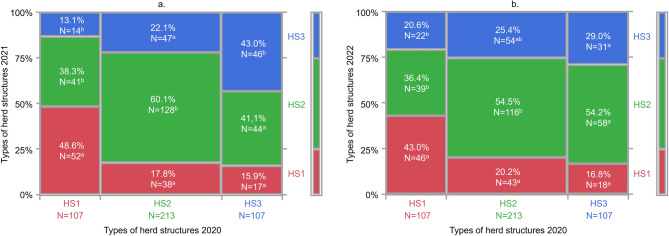
Evolution of herd age structure of farms in (**a**) 2021 and (**b**) 2022 based on their herd age structure in 2020, represented by a mosaic plot. The plots illustrate how farms classified as HS1, HS2, and HS3 in 2020 changed or maintained their herd structure in 2021 (**a**) and 2022 (**b**). Numbers inside the boxes represent the percentage and number of farms in each transition. The table below summarizes the proportion of farms that remained in the same herd structure type over time, based on a contingency table analysis. Different superscripts ^(**a-b**)^ within a row indicate significant differences between proportions within each column (*p* < 0.05). Abbreviations: HS1 (red): Herd structure type 1; HS2 (green): Herd structure type 2; HS3 (blue): Herd structure type 3

The evolution of herd age structure over time is illustrated in Fig. 3 using a mosaic plot and contingency table analysis, which depict how farms transitioned between different types of herd age structures from 2020 to 2022.

Farms classified as HS2 in 2020 showed the greatest herd parity structure stability, with 60.1% of these farms remaining HS2 in 2021 and 54.5% in 2022. Conversely, HS1 farms in 2020 presented moderate stability, with 48.6% staying in the same group in 2021 and 43% in 2022. Additionally, it is noteworthy that 38.3% of HS1 farms in 2020 switched to HS2 in 2021, and 36.4% did so in 2022.

Lastly, HS3 farms in 2020 were the least stable in terms of herd parity structure, with 43% of the farms remaining in this group in 2021, and only 29% in 2022. From HS3 farms in 2020 the 41.1% switched to HS2 in 2021 and 54.2% did so in 2022.

Regarding the degree of agreement between the types of herd age structure in different years, the Kappa coefficient (κ) between 2020 and 2021 was 0.248 (*p* < 0.01), indicating a fair level of agreement, while the coefficient κ was 0.124 (*p* < 0.01) between 2020 and 2022, showing lower agreement and suggesting that the stability of the herd structure decreases over time.

## Discussion

This study addresses the evolution of census structure over time and how productive variables related to farm efficiency are affected and evolve. The classification of farms into three types of herd age structure according to a quadratic function of census distribution by parities has been previously proposed by Sanz-Fernández et al. [15] in a one-year study without a longitudinal evaluation. This classification provides relevant information about census dynamics over the three years studied. However, this classification relies on predefined group sizes based on the coefficient “a” of the quadratic function for each farm, following a fixed 25-50%-25% distribution. Alternative classification methods, such as regression trees or clustering algorithms, deserve to be explored in future studies to improve objectivity and flexibility of this classification, allowing for a more precise representation of the biological variability across farms.

The results from the repeated measures analysis indicate a significant variation of the productive parameters over the three years of the study. In general, there was an improvement of the productivity parameters between 2020 and 2021, with an increase in prolificacy (TB and BA piglets per parity) and a better farrowing rate. The highest mean annual productivity (across all farms) was reached in 2021, with 30.24 PWSY. These results would correspond to average high-productivity sows [23], and their evolution up to 2021 aligned well with that observed for the whole BDporc farms dataset [24] with a mean increase of 4.2 PWSY in the Spanish farms during 10 years (from 2010 to 2020).

In parallel to the increase in prolificacy, the current results also show a worrying increase in mortality during lactation (+ 1.63%) over the three years studied. This decrease in the survivability of piglets is possibly derived from the increase in the litter size of hyperprolific sows [2, 25], which greatly concerns the pig sector [26].

However, in 2022, despite the increase in prolificacy regarding previous years, the progressive increase in farm productivity was halted, with an average below 30 PWSY; which represented returning productivity levels similar to the Spanish average in 2018 [27]. Likewise, other reproductive variables, such as farrowing rate, percentage of sows returning to oestrus, or the yearly number of farrowings, also showed worse average results in 2022. These results are probably related to the serious outbreaks reported since 2020 in the northwest of Spain, involving a strain of porcine reproductive and respiratory syndrome virus (PRRSV-1 or Betaarterivirus suid 1, subtype-1), known as Rosalía [28]. That PRRSV strain is characterized by its higher virulence [29, 30], and spread to the rest of Spain during 2021 and 2022, considerably increasing the rates of abortions, SB, and piglet mortality during lactation [31]. This could explain the observed loss of productivity, as well as the high rates of mortality during lactation. It is expected that these parameters will improve and keep following the upward trend in Spain’s production in previous years [26, 32].

The interaction between the herd age structure and the variation over the years was not significant for the main productive variables. This indicates that the temporal evolution of variables such as PWSY, farrowings per sow and year, and average prolificacy were similar across the different herd age structure types (HS1, HS2, HS3). In other words, the differences between the farm types were maintained over time, suggesting that the herd age structure defined in 2020 continued to have a consistent impact on productivity in 2021 and 2022, without significant fluctuations due to the interaction with time. This highlights the importance of herd age structure as a factor of productivity and that maintaining an appropriate census structure (such as HS1) over time can help to prevent productivity fluctuations [7].

When considering the different types of herd age structure in 2020, without considering the temporal factor, differences between the three established types of structure were reported for most productive variables, reinforcing the message that some census structures could be more favourable than others in terms of (re)production efficiency.

Thus, when considering the different types of herd age structure in 2020 (HS1, HS2, and HS3), and based on the initial hypothesis that, with a current average of around 2.43 farrowings per sow and year, the census structure of one year can drastically change its dynamics from one to two years; a farm with HS1 in 2020, with a high proportion of sows between the 3rd and 5th parities (around 45% of the census), and achieving the best productive results in 2020, with an average of 31.45 PWSY, can transition to HS3, with a higher proportion of old sows, which are less productive, in just one year.

In this regard, farms with HS1 in 2020 presented superior performance in terms of annual productivity, farrowing rate, average prolificacy (TB and BA), and W piglets, in the subsequent years, 2021 and 2022. This finding indicates that a balanced census with a concave-downward trend curve and a higher proportion of sows in intermediate parities is associated with higher production efficiency, not only in a specific year as stated by Sanz-Fernández et al. [15], but also over the medium term. However, it is worth noting that while sows in their 3rd to 5th parity are associated with higher BA piglets, second parity sows have lower piglets mortality rates during lactation, thus leading to superior weaning performance [2]. These results suggest that an optimal herd age structure should ensure a balanced proportion of sows, minimizing sow losses up to the 5th parity, as suggested by De Andrés et al. [18] for a herd structure similar to HS1. Therefore, this superior performance of HS1 compared to HS2 and HS3 farms could be attributed to a better sow reproduction management, which would minimize early culling (79% of the sow census up until the 5th parity) and lead to an increase in mean sows longevity, thus harnessing the full productive potential of these sows, as reflected in shorter intervals between weaning and conception. Nevertheless, these variables (WSI, WOI, WCI) exhibited high kurtosis, indicating deviations from a normal distribution, so results should be interpreted with caution. Future research could explore alternative modeling approaches better suited to account for non-normal distributions, potentially improving the accuracy of productivity analyses.

Additionally, related to good management of sows and their longevity, HS1 farms had a lower percentage of first-cycle sows (about 17–18% of the census over the three years), implying lower replacement, thus making them more profitable farms [33].

In contrast, farms with HS2 in 2020, which had a trend curve close to a straight line and are aligned with the herd age structure proposed by various authors as the ideal census structure [34–36], presented intermediate productivity results in 2021 and 2022, lower than those of HS1. Finally, farms with HS3 in 2020, with an upward concave trend, achieved the worst results in the following years. This poor performance probably derived from the unbalanced census structure; these farms had a high proportion (> 26%) of sows with 6 or more parities over the three years, a stage when sow productivity decreases.

As a final point, the analysis of the evolution of herd age structure showed that, although some farms maintained their census structure, many others made significant changes in their population dynamics across time. What is more, the low level of agreement between census structures in consecutive years reflects the difficulty of keeping a stable herd age structure in the medium term, as had been previously stated by Koketsu [7]. The observed variability in the dynamics of commercial sow herds in the current study had also been reported by Rodríguez-Sánchez et al. [10].

In summary, farms classified as HS2 in 2020 were the most stable over time, with over 60% of farms maintaining this structure in 2021 and over 50% in 2022. The constant loss of sows per parity of this type of structure, with an average decrease of 2% of sows from one parity to another, enabled the maintenance of a stable census over time [34–40]. In contrast, HS3 farms were the least stable, with a high proportion of them transitioning to HS2 in the subsequent years; probably because of a high replenishment to compensate for the aging census. On the contrary, HS1 farms, despite achieving the best productivity results, maintained moderate stability in the following two years, with 48.6% of farms remaining as HS1 in 2021 and 43% in 2022.

To improve the stability of HS1 structure, associated with better productivity levels, it is suggested to follow the criteria proposed by Vizcaíno et al. [41], minimizing the loss of sows in first cycles; thus increasing the productive life of sows and having more sows in intermediate parities to achieve a more productive census distribution. The maintenance of this structure over time should be combined with the criterion of eliminating sows based on their performance and removing a higher proportion of sows from the 5th parity onwards. Implementing these criteria would reduce the necessary replacement percentage and increase the productive life of sows, reducing the costs of their depreciation [42].

## Conclusions

The classification of farms according to the quadratic function of census distribution allowed for the differentiation of three types of herd age structure and facilitated a better understanding of how census distribution at each parity evolves and conditions farm productivity over time.

It is not easy to maintain the herd age structure in a farm. However, it is advisable to have HS1 structure, with a downward-concave trend. These HS1 farms had the best productivity results maintained over time, highlighting the importance of having a high proportion of sows in intermediate parities. The HS1 census distribution could be recommended as the optimal herd age or parity structure from a productive standpoint in the short and medium term, provided that an adequate replacement policy is maintained to ensure its census stability in the future. Specific sow replacement criteria based on their performance, but avoiding early elimination, and harnessing the productive potential of sows up to the 5th parity, help to gain stability in the maintenance of this optimal herd age structure. In addition, further research should focus on identifying specific management factors to optimize productivity and ensure the long-term stability of the HS1 structure.

## Data Availability

No datasets were generated or analysed during the current study.

## Abbreviations

BA: Piglets born alive
HS1: Herd structure type 1
HS2: Herd structure type 2
HS3: Herd structure type 3
PWSY: Number of piglets weaned per sow and year
RMSE: Root mean square error
SB: Stillborn piglets
TB: Total piglets born
W: Weaned piglets
WCI: Weaning to conception interval
WOI: Weaning-to-oestrus interval
WSI: Weaning-to-first-service interval
